# How to Decide the Number of Gait Cycles in Different Low-Pass Filters to Extract Motor Modules by Non-negative Matrix Factorization During Walking in Chronic Post-stroke Patients

**DOI:** 10.3389/fnhum.2022.803542

**Published:** 2022-04-06

**Authors:** Yuta Chujo, Kimihiko Mori, Tomoki Kitawaki, Masanori Wakida, Tomoyuki Noda, Kimitaka Hase

**Affiliations:** ^1^Department of Physical Medicine and Rehabilitation, Kansai Medical University Hospital, Hirakata, Japan; ^2^Department of Physical Medicine and Rehabilitation, Kansai Medical University, Hirakata, Japan; ^3^Faculty of Rehabilitation, Kansai Medical University, Hirakata, Japan; ^4^Department of Mathematics, Kansai Medical University, Hirakata, Japan; ^5^Brain Information Communication Research Laboratory Group, Advanced Telecommunications Research Institutes International (ATR), Kyoto, Japan

**Keywords:** motor module, non-negative matrix factorization, low-pass filter, gait cycle, gait, post-stroke patient

## Abstract

The motor modules during human walking are identified using non-negative matrix factorization (NNMF) from surface electromyography (EMG) signals. The extraction of motor modules in healthy participants is affected by the change in pre-processing of EMG signals, such as low-pass filters (LPFs); however, the effect of different pre-processing methods, such as the number of necessary gait cycles (GCs) in post-stroke patients with varying steps, remains unknown. We aimed to specify that the number of GCs influenced the motor modules extracted in the consideration of LPFs in post-stroke patients. In total, 10 chronic post-stroke patients walked at a self-selected speed on an overground walkway, while EMG signals were recorded from the eight muscles of paretic lower limb. To verify the number of GCs, five GC conditions were set, namely, 25 (reference condition), 20, 15, 10, and 5 gate cycles with three LPFs (4, 10, and 15 Hz). First, the number of modules, variability accounted for (VAF), and muscle weightings extracted by the NNMF algorithm were compared between the conditions. Next, a modified NNMF algorithm, in which the activation timing profiles among different GCs were unified, was performed to compare the muscle weightings more robustly between GCs. The number of motor modules was not significantly different, regardless of the GCs. The difference in VAF and muscle weightings in the different GCs decreased with the LPF of 4 Hz. Muscle weightings in 15 GCs or less were significantly different from those in 25 GCs using the modified NNMF. Therefore, we concluded that the variability extracted motor modules by different GCs was suppressed with lower LPFs; however, 20 GCs were needed for more representative extraction of motor modules during walking in post-stroke patients.

## Introduction

The human body has numerous joints and muscles. How the central nervous system controls the musculoskeletal system, which has the highest redundancy during human movements, has long been known as a problem (Bernstein, 1967). Researchers try to solve this problem based on the muscle synergy theory that the brain does not control each muscle independently but a few motor modules in the spinal cord to execute complex movements. The motor modules were identified by mathematical processing (e.g., non-negative matrix factorization [NNMF], factor analysis, principal component analysis, and independent component analysis) from surface electromyography (EMG) data during movement tasks. In particular, the identification of motor modules using the NNMF algorithm is implemented in neural networks because the non-negativity constraints are similar to motor control in that the synapses are either excitatory or inhibitory (Lee and Seung, 1999). A previous study has provided evidence that the data using the NNMF algorithm were not contingent artifacts but reflected structures of the motor modules (Tresch et al., 2006). Recently, these methods have been used in functional assessment and therapeutic effects in post-stroke patients (Bowden et al., 2010; Clark et al., 2010; Cheung et al., 2012; Routson et al., 2013).

In general, to remove the variability of muscle activity in each gait cycle (GC) or the noise due to the artifact, identifying motor modules from EMG signals involves the following pre-processing steps: the choice of GCs, or high-pass filter, rectified, low-pass filter (LPF), and normalization. Oliveira et al. (2014) have shown that sufficient GCs provided the extracted motor modules with higher quality in healthy participants (Oliveira et al., 2014). Other studies have previously suggested that the required GCs to extract the ideal envelope waveform in healthy participants were not less than 6–10 (Shiavi et al., 1998) or 20 (Gabel and Brand, 1994). Thus, fewer GCs may affect the extraction of motor modules, similar to filter and normalization. It is still unclear how many GCs are sufficient to effectively remove the variability of EMG signals and to extract the motor modules consistently in post-stroke patients. In fact, there are no standard criteria for the number of GCs necessary for the extraction of motor modules in post-stroke patients. Previous studies have inconsistently showed that the measured GCs were 10 (Barroso et al., 2014, 2017) or were not defined, such as walking distance and duration (Neptune et al., 2009; Bowden et al., 2010; Clark et al., 2010; Hashiguchi et al., 2016). Hashiguchi et al. (2016) reported that a limited number of GCs might affect the extraction of motor modules as a limitation in such studies. The determination of representative extracted motor modules, even with a small number of GCs and minimum-required GCs, is an important issue in clinical settings because post-stroke patients with poor walking performance are not able to walk long distances and have increased step-by-step variability (Balasubramanian et al., 2009).

Meanwhile, previous studies have shown that differences in LPFs affected the number of motor modules, the explained variance (e.g., variability accounted for [VAF] or cumulative explained variance), and muscle weightings. Moreover, high LPFs increase the variability of module extraction because retaining the high frequency content in the EMG signals would decrease the total VAF. It is clear that the LPFs affect the extraction of motor modules in healthy participants and in patients with cerebral palsy; however, this remains unclear in post-stroke patients.

In this study, we investigated how the number of GCs influenced the extracted motor modules in post-stroke patients. Since the EMG signals might be highly variable for the post-stroke patients with high variability in performance, it was necessary to confirm the influence of variability on the extraction of motor modules in the consideration of pre-processing, such as the GCs selection and the LPFs. Thus, our study might be useful in comparing previous studies and help apply the methodology to extract muscle modules more consistently.

## Materials and Methods

### Participants

This study included ten participants with chronic post-stroke hemiparesis. The inclusion criteria were as follows: (i) at least 6 months since single unilateral stroke onset and (ii) ability to walk independently on a level surface using a cane. The exclusion criteria were lower extremity joint pain, contractures, and neurological or musculoskeletal disorders other than stroke that affect walking.

All participants provided informed consent according to the Declaration of Helsinki, and the study protocol was approved by the ethics committee of the institution (Kansai Medical University #2019148).

### Experiment Protocol

The participants repeatedly walked at a self-selected speed with a cane on a 7-m-long over-ground walkway: 1 m for acceleration, followed by 5 m for gait measurement, and 1 m of deceleration. Notably, half of the participants used an ankle foot orthosis (AFO) daily, but, in this study, they walked without an AFO. Gait measurements were performed on the level surface until at least 25 GCs could be completed, excluding obvious outliers (e.g., acceleration, deceleration, turning, and significant noise). Muscle activity during walking was recorded using EMG signals, and foot switch was recorded using the Noraxon Clinical DTS system (Scottsdale, AZ, United States; sampling rate: 1,500 Hz). The EMG activity was recorded from the gluteus medius (Gmed), gluteus maximus (Gmax), rectus femoris (RF), vastus medialis (VM), semitendinosus (ST), tibialis anterior (TA), gastrocnemius lateralis (GCL), and soleus (SOL) of the affected side using superficial bipolar Ag–AgCl electrodes (Blue sensor; Medicotest, Inc., Olstykke, Denmark) with an inter-electrode spacing of 2 cm. The skin was cleaned using an alcohol swab to minimize impedance, and the electrodes were placed according to the recommendations for EMG signals for the non-invasive assessment of muscles (Hermens et al., 2000). A foot switch was placed under the affected foot to identify initial contacts during walking. The data were analyzed using MATLAB R2018a (The MathWorks, Inc., Natick, MA, United States). To verify the number of GCs or the cut-off of the filtering frequency in different pre-processing methods, which influenced the representative extraction and reconstruction of motor modules for walking, five conditions for GCs were set, which were as follows: 25 GCs vs. 20 GCs, 15 GCs, 10 GCs, and 5 GCs, in three LPF conditions with reference to previous studies: 4, 10, and 15 Hz (Clark et al., 2010; Monaco et al., 2010; Routson et al., 2013; Hashiguchi et al., 2016). The number of GCs from the beginning for each cycle was determined in all participants.

### Non-negative Matrix Factorization

**FIGURE 1:**
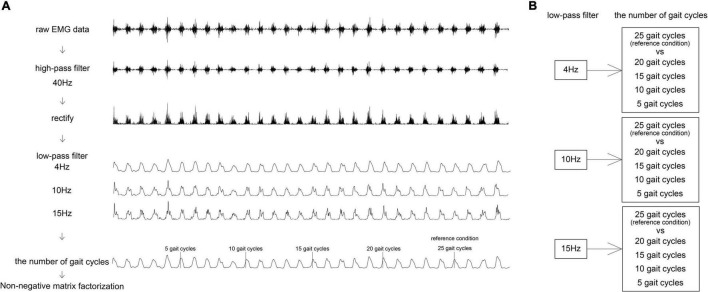
A flowchart of pre-processing for NNMF. Algorithm **(A)** and condition setting **(B)**. **(A)** Pre-processing of one muscle. NNMF, non-negative matrix factorization.

The EMG signals were high-pass filtered (40 Hz) with a zero-lag second-order Butterworth filter (Bowden et al., 2010; Clark et al., 2010; Routson et al., 2013), demeaned, rectified, low-pass filtered (4, 10, and 15 Hz) with a zero-lag second-order Butterworth filter, and then the number of GCs was determined (Figure 1). The initiation of each GC was determined as the initial contact by foot-switch data. One GC duration was resampled at 101 data points. To compare between participants and among different GCs, the EMG signals amplitude divided by the peak value of each muscle during walking was normalized in each condition (Bowden et al., 2010; Clark et al., 2010; Routson et al., 2013). For each participant, the normalized EMG signals were represented as an m × t matrix (original EMG: oEMG), where m indicates the number of muscles (eight in this study), and t is the time course (the number of GCs × 101 data points: the concatenation of a given number of GCs) (Bowden et al., 2010; Clark et al., 2010; Routson et al., 2013). The oEMG was separated into muscle weightings and activation timing profiles using the NNMF algorithm (Lee and Seung, 1999) of the MATLAB function “nnmf”; options used were default values. These are represented by the following equations:


oEMG=W×H+e

W×H=rEMG,

Where, W is m × n matrix (n is the number of modules), H is an n × t matrix, and rEMG is m × t matrix reconstructing the EMG. The NNMF algorithm first ran two random matrices, W and H. The EMG signals were optimized by an iteratively performed optimization process until it converged to a minimized error. The output normalization method of motor modules was by maximum value of each W (Safavynia and Ting, 2012; Chvatal and Ting, 2013).

In the conventional NNMF algorithm described above, since it was decomposed into muscle weightings and activation timing profiles, muscle weightings were affected when activation timing profiles changed. Muceli et al. (2010) unified the muscle weightings between conditions to confirm differences in activation timing profiles. Comparable with the previous study, in our study, we developed a modified NNMF algorithm, which unifies activation timing profiles between GC conditions as one in each module to confirm slight differences in muscle weightings. We performed modified NNMF in the LPF of 4 Hz, representing the motor modules in any GC. Thus, to compare the muscle weightings among different GC conditions in more detail, we performed additional analysis. The modified NNMF algorithm was performed using the same MATLAB function “nnmf” as the conventional NNMF algorithm (Supplementary File 1). These are represented by the following equation:


datasets(oEMG)5GCs,10GCs,15GCs,20GCs,25GCs=W ×5GCs,10GCs,15GCs,20GCs,25G⁢C⁢sH.

This dataset consisted of averaging different numbers of GCs. For each participant, the datasets were represented as an m’ × t’ matrix (40 × 101 matrix), where m’ indicates the number of muscles (8 muscles × 5 GC conditions), and t’ is the time course (101 data points: the average of a given number of GCs).

### Determining the Number of Modules

An adequate number of modules were not *a priori* assumption. Therefore, we assumed that the adequate number of modules ranged from 1–8. To determine the adequate number to rEMG, we calculated the VAF, which is the ratio of the sum of the squared error (oEMG – rEMG)^2^ to the sum of the squared oEMG; oEMG^2^. These are represented by the following equation:


VAF=[1-(oEMG-rEMG)/2oEMG]2×100.

Variability accounted for is the similarity between the oEMG and rEMG. We calculated the VAF in the total muscles (VAF_*total*_) and each muscle (VAF_*muscle*_). Since the criteria of VAF affected the determination of the number of modules, we made the same criteria in all conditions. We selected two criteria for the optimal number of modules in reference to a previous study (Clark et al., 2010). First, the number of modules did not increase if 90% VAF_*total*_ was achieved. Second, the number of modules did not increase if 90% VAF_*muscle*_ was achieved, and if VAF_*muscle*_ with the lowest value did not increase by 5% or more than the previous VAF_*muscle*_.

### Comparison of Muscle Weightings

If the number of modules were different for each participant, it would have been difficult for us to assess the module quality between participants. Thus, to compare the changes in module quality between participants and among different GCs, the number of modules was set to four (Clark et al., 2010; Routson et al., 2013). By using this method, the muscle weightings in each module were comparable. Muscle weighting discrimination was performed, as previously described (Neptune et al., 2009; Clark et al., 2010; Gizzi et al., 2011; Routson et al., 2013).

### Statistical Analysis

All statistical analyses were performed using the R software version 3.6.2. Different GCs in the number of modules, VAF from 1 to 7 (VAF_8_ was excluded because it is 100%), and muscle weightings were assessed. The differences between conditions (the number of GCs) were compared using the Friedman test (significance level α = 0.05). The *post hoc* analyses of GCs, in which 25 GCs were set as a reference condition, were carried out using the Wilcoxon signed-rank sum test with Bonferroni’s correction for multiple comparisons (four comparisons resulting in the significance level α = 0.05/4 = 0.0125). The effect size was calculated as *r* = Z-stat/√N.

## Results

The study comprised of 10 post-stroke patients (age: 69.6 ± 7.2 years; height: 161.8 ± 11.6 cm; weight: 59.8 ± 11.2 kg; sex: 7 men and 3 women; paretic side; 6 right and 4 left; the score of motor paralysis (Fugl-Meyer Assessment of Lower Extremity): 26.3 ± 3.9; walking speed: 0.46 ± 0.1 m/s) (Table 1).

**TABLE 1:** Participant’s characteristics.

Patient	Sex	Diagnosis	Paretic side	Age, yr	Months since event	FMA-motor	FMA-sensory	FMA-synergy	Walk speed, m/sec	MAS	Daily use of an AFO
1	Male	hemorrhage	Right	79	42	24	11	16	0.32	1	+
2	Male	hemorrhage	Left	68	12	22	6	14	0.46	1	+
3	Female	infarction	Right	68	88	25	12	17	0.43	1.5	
4	Male	hemorrhage	Left	76	24	28	12	22	0.51	1	
5	Female	hemorrhage	Right	75	242	21	7	13	0.46	1.5	
6	Female	hemorrhage	Right	59	8	32	12	21	0.54	1	+
7	Male	infarction	Left	69	204	33	12	22	0.52	3	
8	Male	infarction	Right	77	11	27	12	19	0.59	1.5	
9	Male	infarction	Left	58	6	27	12	16	0.37	1	+
10	Male	infarction	Right	67	6	24	12	15	0.41	1.5	+

*FMA, Fugl-Meyer Assessment of Lower Extremity; MAS, Modified Ashworth Scale; AFO, ankle foot orthosis.*

### Number of Modules

**FIGURE 2:**
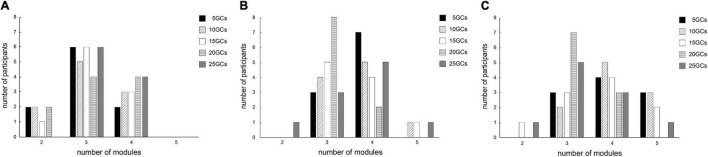
The effects of different conditions on the number of extracted modules by NNMF. The effects of different GCs when the LPFs were 4 Hz **(A)**, 10 Hz **(B)**, and 15 Hz **(C)**. NNMF, non-negative matrix factorization; LPF, low-pass filter; GC, gait cycle.

The number of modules was 2–5 for all conditions. The different GCs did not influence the number of modules when the LPFs were 4 and 10 Hz (*p* = 0.35 and *p* = 0.08, respectively; Figures 2A,B). In contrast, the number of modules on conditions where the LPF was 15 Hz was significantly influenced by different GCs (χ^2^ = 14.29, *p* = 0.006, Figure 2C). However, there were no significant differences in the *post hoc* analysis, although the number of modules at 5 GCs and 10 GCs tended to be more than 25 GCs (*p* = 0.047 and *p* = 0.042, respectively).

### Variability Accounted for

**FIGURE 3:**
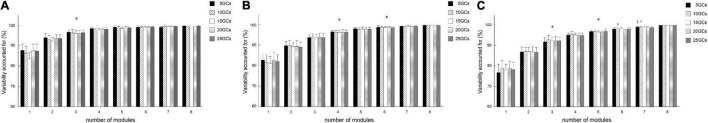
Effects of different conditions on total variability accounted for. Effects of different GCs when the LPFs were 4 Hz **(A)**, 10 Hz **(B)**, and 15 Hz **(C)**. The bars of total variability accounted for indicating the conditional median, and the error bars indicate the median absolute deviations. Significant differences are marked with *(*p* < 0.05 in the Friedman test only) and †(*p* < 0.05 in the Friedman test, and *p* < 0.0125 when comparing the reference condition, 25 GCs, or *p* < 0.016 when comparing between LPFs in the Bonferroni-corrected Wilcoxon signed-rank sum test). LPF, low-pass filter; GC, gait cycle.

The different GCs partially influenced VAF under all conditions of the LPFs (Figures 3A–C).

### Muscle Weightings and Activation Timing Profiles

Module 1 was mainly formed by leg extensor muscles, which are Gmed, Gmax, RF, and VM, during early stance, and ST, GCL, and SOL were slightly activated. Module 2 was formed by the ankle plantar flexor, which is the GCL and SOL, during the late stance. Module 3 was formed by TA and RF slightly during early stance and early swing. Module 4 was mainly formed by ST during the late swing and early stance. TA in module 3 and ST in module 4 were the maximum values on average because for all participants in our study, TA was the most weighting in module 3 and ST was the most weighting in module 4.

**FIGURE 4:**
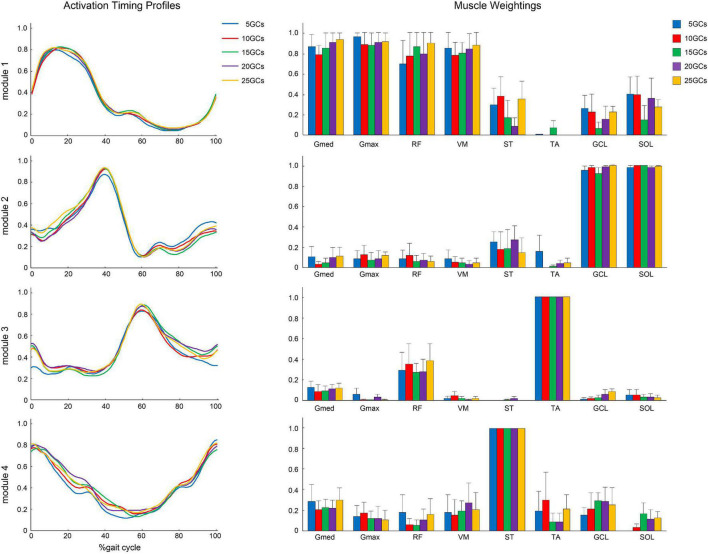
The activation timing profiles and muscle weightings of assuming four modules. The effect of different GCs when the LPF was 4 Hz. Activation timing profiles are shown as the conditional mean. The bars of muscle weightings indicate the conditional median, and the error bars indicate the median absolute deviations. LPF, low-pass filter.

**FIGURE 5:**
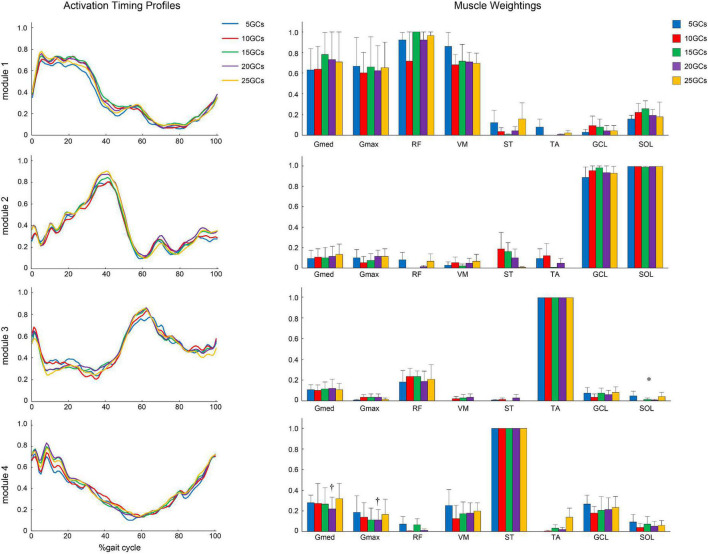
The activation timing profiles and muscle weightings of assuming four modules. The effect of the different GCs when the LPF was 10 Hz. Activation timing profiles are shown as the conditional mean. The bars of muscle weightings indicate the conditional median, and the error bars indicate the median absolute deviations. Significant differences are marked with * (*p* < 0.05 in the Friedman test only) and † (*p* < 0.05 in the Friedman test, and *p* < 0.0125 when comparing the reference condition, 25 GCs in the Bonferroni-corrected Wilcoxon signed-rank sum test). LPF, low-pass filter; GC, gait cycle.

**FIGURE 6:**
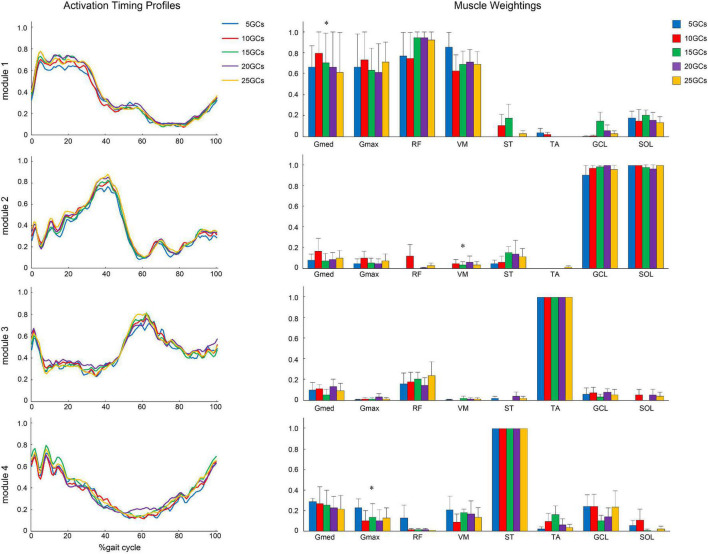
The activation timing profiles and muscle weightings of assuming four modules. The effects of different GCs when the LPF was 15 Hz. Activation timing profiles are shown as the conditional mean. The bars of muscle weightings indicate the conditional median, and the error bars indicate the median absolute deviations. Significant differences are marked with * (*p* < 0.05 at the Friedman test only). LPF, low-pass filter.

The different GCs did not influence the muscle weightings when the LPF was 4 Hz (Figure 4). In contrast, the different GCs partially influenced muscle weightings when the LPFs were 10 and 15 Hz (Figures 5, 6). In module 4, when the LPF was 10 Hz, Gmed and Gmax at 20 GCs were significantly decreased than in the reference condition (25 GCs) (*r* = 0.98, *p* = 0.002, and *r* = 0.79, *p* = 0.013, respectively, Figure 4). However, no significant differences were observed among GCs in the *post hoc* analysis at 15 Hz (Figure 6). The higher the frequency of LPFs, the more was the noise in the activation timing profiles (as shown in the left columns in Figures 4–6).

**FIGURE 7:**
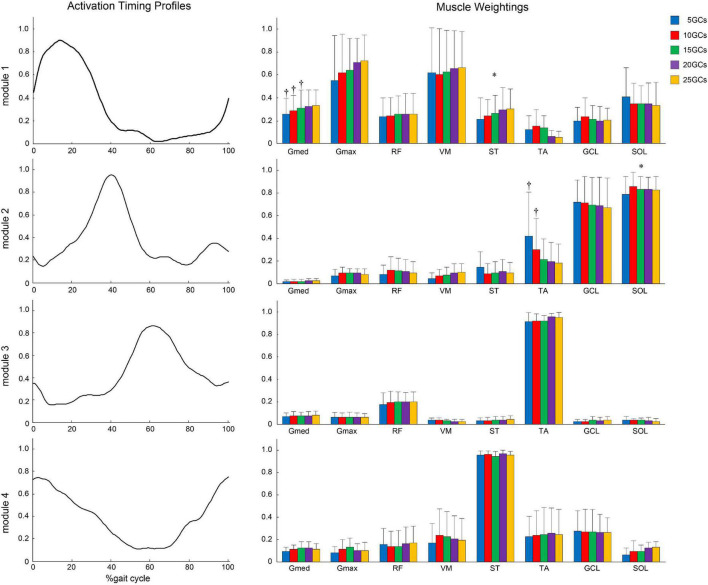
Same activation timing profiles among different GCs and muscle weightings. The effects of the different GCs when the LPF was 4 Hz. Activation timing profiles are shown as the conditional mean. The bars of muscle weightings indicate the conditional median, and the error bars indicate the median absolute deviations. Significant differences are marked with * (*p* < 0.05 in the Friedman test only) and † (*p* < 0.05 in the Friedman test, and *p* < 0.0125 when comparing the reference condition, 25 GCs in the Bonferroni-corrected Wilcoxon signed-rank sum test). LPF, low-pass filter; GC, gait cycle.

In the modified NNMF algorithm, assuming the same activation timing profiles among different GCs, the different GCs influenced the muscle weightings (Figure 7). In module 1, Gmed at 5 GCs, 10 GCs, and 15 GCs was significantly decreased than in the reference condition (25 GCs) (*r* = 0.91, *p* = 0.005, *r* = 0.82, *p* = 0.004, and *r* = 0.98, *p* = 0.002, respectively). In module 2, TA at 5 GCs and 10 GCs was significantly increased than in the reference condition (*r* = 0.82, *p* = 0.009 and *r* = 0.82, *p* = 0.009, respectively).

## Discussion

### Number of Gait Cycles Necessary to Extract Representative Motor Modules

The variability among GCs was increased in post-stroke patients with poor walking performance (Balasubramanian et al., 2009). Many GCs might be needed to extract the representative modules during walking in post-stroke patients when the variability of the EMG signals in each GC was considered. Previous studies reported that the measured GCs in post-stroke patients were fixed to ten repetitions (Barroso et al., 2014, 2017), or were determined by other criteria, such as walking distance and duration (Neptune et al., 2009; Bowden et al., 2010; Clark et al., 2010; Hashiguchi et al., 2016). These studies did not mention the required number of GCs. Here, we randomly divided 25 GCs into 5, 10, 15, 20, and 25 GCs, then, a minimum number of clinically measurable GCs were selected as the representative for the extraction of motor modules using NNMF during over-ground walking because of the variability from low endurance and poor performance for post-stroke patients, and the spatial limitations of clinical setting.

The number of modules did not significantly change by the different GCs in each LPF, although the number of modules at 5 and 10 GCs tended to be smaller than those at 25 GCs on the LPF of 15 Hz (Figure 2C). Similarly, the VAF at 5 and 10 GCs was significantly smaller than that at 25 GCs on the LPF of 15 Hz (Figure 3C). This indicates that a few GCs might cause variability in the EMG signals for each step, in addition to noisier EMG signals with high LPFs. The noise removal on the LPF of 4 or 10 Hz would have less effect on the change of the number of modules and the VAF owing to the variability from a few GCs. Our findings suggested that the number of modules and the VAF were representatively extracted at lower LPF frequencies, despite a small number of GCs, such as 5 GCs.

Moreover, the muscle weightings of the motor modules might be affected by the measured GCs if the muscle activity was variable for each GC. Here, the number of modules was set to four to simplify the comparison of the muscle weightings across participants and conditions, which has been widely used in other studies (Clark et al., 2010; Routson et al., 2013; Barroso et al., 2014; Kieliba et al., 2018). The different GCs compared with 25 GCs did not affect the muscle weightings on 4 Hz significantly, whereas these partially significantly affected the muscle weightings on higher LPFs (10 and 15 Hz). These results suggest that muscle weightings were also representatively extracted using a small number of GCs in lower LPFs as well as the number of modules and the VAF. In contrast, it should be noted that the very low LPFs, such as 0.5 Hz, were likely to reproduce poorly because of the oversmoothed signal (Kieliba et al., 2018).

### Modified Muscle Weightings Unified Activation Timing Profiles

While a modified NNMF algorithm, which could “unify muscle weightings” from GC conditions as one, was used to detect a slight change of activation timing profiles between conditions (Clark et al., 2010; Muceli et al., 2010; Gizzi et al., 2011), our modified NNMF algorithm, which “unified activation timing profiles” from GC conditions as one, was to detect a slight change of muscle weighting between GC conditions. In our study, the statistical analyses, which directly compared the muscle weightings between GC conditions, were the same as in a previous study (Kieliba et al., 2018). In conventional NNMF algorithm, there might be no difference in the LPF of 4 Hz in conventional NNMF algorithm because the influence of activation timing profiles between GC conditions was not considered. The slight differences in activation timing profiles influenced muscle weightings in the conventional NNMF algorithm because of “matrix factorization.” The modified NNMF algorithm would enable the confirmation of slight changes in muscle weightings between the intervals of GCs. Our results showed that the muscle weightings in Gmed of module 1 were low in the conditions of 15 GCs or less, and those in the TA of module 2 were high under the conditions of 10 GCs or less. Thus, we found that 20 GCs were needed to extract more representative motor modules. The new method showed that the motor modules using at least 20 GCs were more robustly extracted in the consideration of variability in each step for post-stroke patients on the level ground, even when the noise of EMG signals was minimized. A previous study suggested that at least 20 GCs were needed to account for step-to-step variability even in healthy participants (Oliveira et al., 2014). Although the previous study was used on the LPF of 10 Hz, post-stroke patients in the present study had similar results by adjusting LPF.

### Study Limitations and Future Directions

Some limitations in our study should be noted. First, the sample size was small; therefore, the potential effects of patient characteristics could be considered. Our study would provide gait characteristics of the chronic phase because patients were on an average 64.3 ± 87.8 months from the onset of stroke. Our findings, with respect to the measured GCs, were useful, despite differences in severity and duration from onset. Moreover, walking speed did not affect these results because patients walked at an average speed of 0.46 ± 0.1 m/s. Therefore, future studies should compare healthy participants and/or patients with other diseases to obtain robust results.

Second, methodologically speaking, we selected the first N cycles for each condition in this study. It should also be considered to select the methods for possible combinations of GCs up to *N* randomly. However, the methods in our study were very simple and close to clinical conditions. Moreover, we thought that the method selecting the first *N* cycles was low in bias because the variability in each GC was unpredictable.

Third, the patients walked with assistive devices, such as a cane and/or an AFO, in daily living. The use of a cane compensates for the balance and weight-bearing during the paretic stance, which might reduce the variability between GCs. Meanwhile, removing the AFO might cause instability of the ankle, such as ankle inversion. Although the effects on lower-limb modules, such as motor control with a cane, were confirmed, the effects on conditions without the use of cane require further investigation.

Finally, the influence of pre-processing, except for LPFs, has not been studied in this study. Previous studies suggested that different band-pass filters, EMG normalization methods, and output normalization methods slightly influenced muscle weightings (Banks et al., 2017; Shuman et al., 2017; Kieliba et al., 2018; Ao et al., 2020). In particular, Shuman et al. (2017) suggested that muscle weightings were more similar in different LPF conditions for EMG data normalized to unit variance than peak amplitude in children. In this study, we included the method to choose high-pass filter (40 Hz) and EMG normalization by peak values during walking as many previous studies in post-stroke patients. Although we considered that these pre-processing methods had no significant effects on the conclusion of the present study because we compared GC conditions in different LPF conditions, future studies should detect the needed number of GCs in consideration of all pre-processing for post-stroke patients to obtain robust results.

## Conclusion

We verified that the number of GCs influenced the motor modules extracted in the consideration of LPFs in chronic post-stroke patients. The different GCs did not affect the number of modules but affected the total VAF under high LPF conditions in the conventional NNMF algorithm. In the comparison of muscle weighting, the conventional NNMF algorithm showed that the different GCs with low LPF, such as 4 Hz, were not affected when the module number was fixed at four. However, the modified NNMF algorithm, in which the activation timing profiles of different GCs were unified as one in each module to detect slight changes in muscle weightings, required EMG signals of 20 GCs or more. Therefore, we suggested that the representative motor modules would be extracted with low LPFs despite a small number of GCs; however, 20 GCs are needed for chronic post-stroke patients in general when a more robust methodology is applied.

## Data Availability Statement

The raw data supporting the conclusions of this article will be made available by the authors, without undue reservation.

## Ethics Statement

The studies involving human participants were reviewed and approved by the Kansai Medical University. Written informed consent for participation was not required for this study in accordance with the national legislation and the institutional requirements.

## Author Contributions

YC: conception, organization, execution of the research project, acquisition and processing of the data, design and execution of the statistical analysis, and writing of the first draft of the manuscript. KM and KH: conception, organization, execution of the research project, review, and critique of the manuscript. TK: design, review, critique of the statistical analysis, and critique of the manuscript. MW and TN: execution of the research project, review, and critique of the manuscript. All authors contributed to the article and approved the submitted version.

## Conflict of Interest

The authors declare that the research was conducted in the absence of any commercial or financial relationships that could be construed as a potential conflict of interest.

## Publisher’s Note

All claims expressed in this article are solely those of the authors and do not necessarily represent those of their affiliated organizations, or those of the publisher, the editors and the reviewers. Any product that may be evaluated in this article, or claim that may be made by its manufacturer, is not guaranteed or endorsed by the publisher.
