# Supplementary material for: How to Decide the Number of Gait Cycles in Different Low-Pass Filters to Extract Motor Modules by Non-negative Matrix Factorization During Walking in Chronic Post-stroke Patients
Source: Front Hum Neurosci. 2022 Apr 6;16:803542. doi: 10.3389/fnhum.2022.803542 (PMC9019077; doi:10.3389/fnhum.2022.803542)
Supplement: Supplementary file 1 [file Data_Sheet_1.DOCX]

Supplementary Material

# Supplementary Data

%data-import

aaa1 = xlsread('S1.xlsx');

aa1 = aaa1(:,2:9);

%highpass-filter

N = 2;

sample = 1500;

Lf = 40;

Wn = 2*Lf/sample;

[B,A] = butter(N,Wn,'High') ;

d1 = filtfilt(B,A,aa1);

%de-meaned

DM1=mean(d1(:,1));

DM2=mean(d1(:,2));

DM3=mean(d1(:,3));

DM4=mean(d1(:,4));

DM5=mean(d1(:,5));

DM6=mean(d1(:,6));

DM7=mean(d1(:,7));

DM8=mean(d1(:,8));

D1=[d1(:,1)-DM1,d1(:,2)-DM2,d1(:,3)-DM3,d1(:,4)-DM4,d1(:,5)-DM5,d1(:,6)-DM6,d1(:,7)-DM7,d1(:,8)-DM8,];

%rectified

a1 = abs(D1);

%pading

PD1 = zeros(10,8);

PDa1 = vertcat(PD1,a1,PD1);

%lowpass-filter(4Hz)

N = 2;

sample = 1500;

Lf = 4;

Wn = 2*Lf/sample;

[B,A] = butter(N,Wn,'Low') ;

ddd4 = filtfilt(B,A,PDa1);

%data cut

dd= ddd4(11:length(ddd4)-10,1:8);

%EMG + gait cycle(paretic)

data = [dd aaa1(:,13)];

%find initial contact

IC1 = find(aaa1(:,13)==1);

IC2 = find(aaa1(:,13)==3);

IC3 = find(aaa1(:,13)==5);

IC4 = find(aaa1(:,13)==7);

IC5 = find(aaa1(:,13)==9);

IC6 = find(aaa1(:,13)==11);

IC7 = find(aaa1(:,13)==13);

IC8 = find(aaa1(:,13)==15);

IC9 = find(aaa1(:,13)==17);

IC10 = find(aaa1(:,13)==19);

IC11 = find(aaa1(:,13)==21);

IC12 = find(aaa1(:,13)==23);

IC13 = find(aaa1(:,13)==25);

IC14 = find(aaa1(:,13)==27);

IC15 = find(aaa1(:,13)==29);

IC16 = find(aaa1(:,13)==31);

IC17 = find(aaa1(:,13)==33);

IC18 = find(aaa1(:,13)==35);

IC19 = find(aaa1(:,13)==37);

IC20 = find(aaa1(:,13)==39);

IC21 = find(aaa1(:,13)==41);

IC22 = find(aaa1(:,13)==43);

IC23 = find(aaa1(:,13)==45);

IC24 = find(aaa1(:,13)==47);

IC25 = find(aaa1(:,13)==49);

IC26 = find(aaa1(:,13)==51);

%gait cycles

GC1 = data(IC1(1):IC2(1)-1,1:8);

GC2 = data(IC2(1):IC3(1)-1,1:8);

GC3 = data(IC3(1):IC4(1)-1,1:8);

GC4 = data(IC4(1):IC5(1)-1,1:8);

GC5 = data(IC5(1):IC6(1)-1,1:8);

GC6 = data(IC6(1):IC7(1)-1,1:8);

GC7 = data(IC7(1):IC8(1)-1,1:8);

GC8 = data(IC8(1):IC9(1)-1,1:8);

GC9 = data(IC9(1):IC10(1)-1,1:8);

GC10 = data(IC10(1):IC11(1)-1,1:8);

GC11 = data(IC11(1):IC12(1)-1,1:8);

GC12 = data(IC12(1):IC13(1)-1,1:8);

GC13 = data(IC13(1):IC14(1)-1,1:8);

GC14 = data(IC14(1):IC15(1)-1,1:8);

GC15 = data(IC15(1):IC16(1)-1,1:8);

GC16 = data(IC16(1):IC17(1)-1,1:8);

GC17 = data(IC17(1):IC18(1)-1,1:8);

GC18 = data(IC18(1):IC19(1)-1,1:8);

GC19 = data(IC19(1):IC20(1)-1,1:8);

GC20 = data(IC20(1):IC21(1)-1,1:8);

GC21 = data(IC21(1):IC22(1)-1,1:8);

GC22 = data(IC22(1):IC23(1)-1,1:8);

GC23 = data(IC23(1):IC24(1)-1,1:8);

GC24 = data(IC24(1):IC25(1)-1,1:8);

GC25 = data(IC25(1):IC26(1)-1,1:8);

%resample

NGC1 = resample(GC1,101,length(GC1),0);

NGC2 = resample(GC2,101,length(GC2),0);

NGC3 = resample(GC3,101,length(GC3),0);

NGC4 = resample(GC4,101,length(GC4),0);

NGC5 = resample(GC5,101,length(GC5),0);

NGC6 = resample(GC6,101,length(GC6),0);

NGC7 = resample(GC7,101,length(GC7),0);

NGC8 = resample(GC8,101,length(GC8),0);

NGC9 = resample(GC9,101,length(GC9),0);

NGC10 = resample(GC10,101,length(GC10),0);

NGC11 = resample(GC11,101,length(GC11),0);

NGC12 = resample(GC12,101,length(GC12),0);

NGC13 = resample(GC13,101,length(GC13),0);

NGC14 = resample(GC14,101,length(GC14),0);

NGC15 = resample(GC15,101,length(GC15),0);

NGC16 = resample(GC16,101,length(GC16),0);

NGC17 = resample(GC17,101,length(GC17),0);

NGC18 = resample(GC18,101,length(GC18),0);

NGC19 = resample(GC19,101,length(GC19),0);

NGC20 = resample(GC20,101,length(GC20),0);

NGC21 = resample(GC21,101,length(GC21),0);

NGC22 = resample(GC22,101,length(GC22),0);

NGC23 = resample(GC23,101,length(GC23),0);

NGC24 = resample(GC24,101,length(GC24),0);

NGC25 = resample(GC25,101,length(GC25),0);

GCtotal25GC = (NGC1 + NGC2 + NGC3 + NGC4 + NGC5 + NGC6 + NGC7 + NGC8 + NGC9 + NGC10 + NGC11 + NGC12 + NGC13 + NGC14 + NGC15 + NGC16 + NGC17 + NGC18 + NGC19 + NGC20 + NGC21 + NGC22 + NGC23 + NGC24 + NGC25)/25;

GCtotal20GC = (NGC1 + NGC2 + NGC3 + NGC4 + NGC5 + NGC6 + NGC7 + NGC8 + NGC9 + NGC10 + NGC11 + NGC12 + NGC13 + NGC14 + NGC15 + NGC16 + NGC17 + NGC18 + NGC19 + NGC20)/20;

GCtotal15GC = (NGC1 + NGC2 + NGC3 + NGC4 + NGC5 + NGC6 + NGC7 + NGC8 + NGC9 + NGC10 + NGC11 + NGC12 + NGC13 + NGC14 + NGC15)/15;

GCtotal10GC = (NGC1 + NGC2 + NGC3 + NGC4 + NGC5 + NGC6 + NGC7 + NGC8 + NGC9 + NGC10)/10;

GCtotal5GC = (NGC1 + NGC2 + NGC3 + NGC4 + NGC5)/5;

jj = horzcat(GCtotal25GC,GCtotal20GC,GCtotal15GC,GCtotal10GC,GCtotal5GC);

mm = jj.';

E = mm;

%% NNMF

[W4,H4] = nnmf(E,4);

%% Reconstruct

R4 = W4*H4;

%Synergy4_VAF

G4 = E-R4;

VAF4Gr = (1-sum(G4.^2)/sum(E.^2))*100;

VAF4LoM1 = (1-sum(G4(1,:).^2)/sum(E(1,:).^2))*100;

VAF4LoM2 = (1-sum(G4(2,:).^2)/sum(E(2,:).^2))*100;

VAF4LoM3 = (1-sum(G4(3,:).^2)/sum(E(3,:).^2))*100;

VAF4LoM4 = (1-sum(G4(4,:).^2)/sum(E(4,:).^2))*100;

VAF4LoM5 = (1-sum(G4(5,:).^2)/sum(E(5,:).^2))*100;

VAF4LoM6 = (1-sum(G4(6,:).^2)/sum(E(6,:).^2))*100;

VAF4LoM7 = (1-sum(G4(7,:).^2)/sum(E(7,:).^2))*100;

VAF4LoM8 = (1-sum(G4(8,:).^2)/sum(E(8,:).^2))*100;

VAF4LoM9 = (1-sum(G4(9,:).^2)/sum(E(9,:).^2))*100;

VAF4LoM10 = (1-sum(G4(10,:).^2)/sum(E(10,:).^2))*100;

VAF4LoM11 = (1-sum(G4(11,:).^2)/sum(E(11,:).^2))*100;

VAF4LoM12 = (1-sum(G4(12,:).^2)/sum(E(12,:).^2))*100;

VAF4LoM13 = (1-sum(G4(13,:).^2)/sum(E(13,:).^2))*100;

VAF4LoM14 = (1-sum(G4(14,:).^2)/sum(E(14,:).^2))*100;

VAF4LoM15 = (1-sum(G4(15,:).^2)/sum(E(15,:).^2))*100;

VAF4LoM16 = (1-sum(G4(16,:).^2)/sum(E(16,:).^2))*100;

VAF4LoM17 = (1-sum(G4(17,:).^2)/sum(E(17,:).^2))*100;

VAF4LoM18 = (1-sum(G4(18,:).^2)/sum(E(18,:).^2))*100;

VAF4LoM19 = (1-sum(G4(19,:).^2)/sum(E(19,:).^2))*100;

VAF4LoM20 = (1-sum(G4(20,:).^2)/sum(E(20,:).^2))*100;

VAF4LoM21 = (1-sum(G4(21,:).^2)/sum(E(21,:).^2))*100;

VAF4LoM22 = (1-sum(G4(22,:).^2)/sum(E(22,:).^2))*100;

VAF4LoM23 = (1-sum(G4(23,:).^2)/sum(E(23,:).^2))*100;

VAF4LoM24 = (1-sum(G4(24,:).^2)/sum(E(24,:).^2))*100;

VAF4LoM25 = (1-sum(G4(25,:).^2)/sum(E(25,:).^2))*100;

VAF4LoM26 = (1-sum(G4(26,:).^2)/sum(E(26,:).^2))*100;

VAF4LoM27 = (1-sum(G4(27,:).^2)/sum(E(27,:).^2))*100;

VAF4LoM28 = (1-sum(G4(28,:).^2)/sum(E(28,:).^2))*100;

VAF4LoM29 = (1-sum(G4(29,:).^2)/sum(E(29,:).^2))*100;

VAF4LoM30 = (1-sum(G4(30,:).^2)/sum(E(30,:).^2))*100;

VAF4LoM31 = (1-sum(G4(31,:).^2)/sum(E(31,:).^2))*100;

VAF4LoM32 = (1-sum(G4(32,:).^2)/sum(E(32,:).^2))*100;

VAF4LoM33 = (1-sum(G4(33,:).^2)/sum(E(33,:).^2))*100;

VAF4LoM34 = (1-sum(G4(34,:).^2)/sum(E(34,:).^2))*100;

VAF4LoM35 = (1-sum(G4(35,:).^2)/sum(E(35,:).^2))*100;

VAF4LoM36 = (1-sum(G4(36,:).^2)/sum(E(36,:).^2))*100;

VAF4LoM37 = (1-sum(G4(37,:).^2)/sum(E(37,:).^2))*100;

VAF4LoM38 = (1-sum(G4(38,:).^2)/sum(E(38,:).^2))*100;

VAF4LoM39 = (1-sum(G4(39,:).^2)/sum(E(39,:).^2))*100;

VAF4LoM40 = (1-sum(G4(40,:).^2)/sum(E(40,:).^2))*100;

VAF4 = [VAF4Gr VAF4LoM1 VAF4LoM2 VAF4LoM3 VAF4LoM4 VAF4LoM5 VAF4LoM6 VAF4LoM7 VAF4LoM8 VAF4LoM9 VAF4LoM10 VAF4LoM11 VAF4LoM12 VAF4LoM13 VAF4LoM14 VAF4LoM15 VAF4LoM16 VAF4LoM17 VAF4LoM18 VAF4LoM19 VAF4LoM20 VAF4LoM21 VAF4LoM22 VAF4LoM23 VAF4LoM24 VAF4LoM25 VAF4LoM26 VAF4LoM27 VAF4LoM28 VAF4LoM29 VAF4LoM30 VAF4LoM31 VAF4LoM32 VAF4LoM33 VAF4LoM34 VAF4LoM35 VAF4LoM36 VAF4LoM37 VAF4LoM38 VAF4LoM39 VAF4LoM40];
